# Medical Management of a Meningioma Presenting as an Ischemic Stroke: A Case Report and Review of the Literature

**DOI:** 10.7759/cureus.89975

**Published:** 2025-08-13

**Authors:** Tomotaro Akamatsu, Arihito Tsurumi, Osamu Hososhima, Ryusei Zako, Yuko Tsurumi

**Affiliations:** 1 Neurological Surgery, Tsurumi Hospital, Koga, JPN; 2 Radiology, Tsurumi Hospital, Koga, JPN

**Keywords:** antithrombotic therapy, cerebral artery compression, conservative management, hyperosmolar therapy, large vessel occlusion, medical recanalization, meningioma-induced ischemic stroke, middle cerebral artery occlusion, sphenoid wing meningioma, spontaneous recanalization

## Abstract

An ischemic stroke caused by meningioma-induced arterial compression is exceedingly rare, with few reported cases of large cerebral artery occlusion due to extrinsic tumor mass effect. We report the case of a 76-year-old man who presented with a severe stroke (National Institutes of Health Stroke Scale (NIHSS) score of 20) caused by left middle cerebral artery (MCA) M1 segment occlusion from a sphenoid wing meningioma. Standard reperfusion therapies were not pursued; intravenous thrombolysis was contraindicated due to unknown symptom onset and tumor-associated hemorrhagic risk; mechanical thrombectomy was unlikely to succeed against extrinsic arterial compression; and emergent tumor resection posed excessive surgical risk given the patient’s age, extensive infarction, and cerebral edema. Instead, intensive medical therapy was initiated, consisting of triple antiplatelet therapy (aspirin, clopidogrel, and ozagrel) and osmotic therapy with intravenous glycerol. Under this regimen, his condition stabilized, and follow-up MR angiography (MRA) at one week demonstrated reestablished flow in the previously occluded MCA, indicating spontaneous recanalization without any surgical or endovascular intervention. The infarct remained localized without hemorrhagic transformation. Given his advanced age, infarct burden, and favorable recovery, elective tumor resection was deferred. He underwent intensive rehabilitation and gradually improved to a modified Rankin Scale (mRS) score of four at discharge and an mRS score of two at one-year follow-up, without recurrent stroke.

To our knowledge, this is one of the few reported cases of meningioma-related large-artery stroke managed successfully with medical therapy alone. Notably, this is the first documented case in which such treatment led to spontaneous MCA recanalization. This case highlights the potential for aggressive medical management to obviate emergent neurosurgery in select patients. Further study is warranted to identify candidates most likely to benefit from such an approach.

## Introduction

An ischemic stroke directly caused by an intracranial neoplasm is exceedingly rare. Meningiomas are among the most common primary brain tumors, yet stroke attributable to a meningioma occurs in only approximately 0.2% to 0.4% of cases [1, 2].

Owing to the rarity of this presentation, no established guidelines currently exist for managing acute ischemic stroke caused by an intracranial tumor. Furthermore, standard acute stroke therapies are usually contraindicated in this scenario. Intravenous thrombolysis (tPA) is typically avoided due to the tumor’s presence (with its attendant hemorrhagic risk) and often an unknown time of stroke onset. Likewise, mechanical thrombectomy is generally not feasible when the artery is occluded by extrinsic compression rather than an intraluminal clot.

In prior case reports, management has most often entailed urgent surgical intervention (tumor resection or bypass surgery) in an attempt to restore cerebral perfusion. Conversely, a purely medical management strategy has been described in only a few instances [3], without enough evidence to establish its effectiveness. This paucity of experience highlights a significant knowledge gap regarding the optimal acute treatment for meningioma-related stroke. Specifically, it remains unclear whether intensive medical therapy alone can achieve vessel recanalization and favorable outcomes in the absence of surgical decompression.

We present a rare case of acute left M1 middle cerebral artery (MCA) occlusion caused by a sphenoid wing meningioma that was successfully managed with intensive medical therapy instead of immediate surgery. This case is one of the few managed without emergent surgery, and to our knowledge, it is the first to demonstrate recanalization of the occluded artery through medical treatment alone. We propose that aggressive medical therapy may obviate the need for high-risk acute surgery in select patients.

## Case presentation

A 76-year-old right-handed man was found in the early morning with acute onset of speech disturbance and right-sided paralysis, which was consistent with a wake-up stroke (he was last seen normal the night before). On arrival, his blood pressure was 178/91 mmHg, and he had an impaired level of consciousness. Neurologically, he exhibited a profound left hemispheric syndrome: a leftward gaze preference with inability to gaze to the right, global aphasia (no response to verbal commands), right homonymous hemianopia, dense right hemiplegia, and right hemispatial neglect. His National Institutes of Health Stroke Scale (NIHSS) score was 20, indicating severe left MCA syndrome [4].

**Figure 1:**
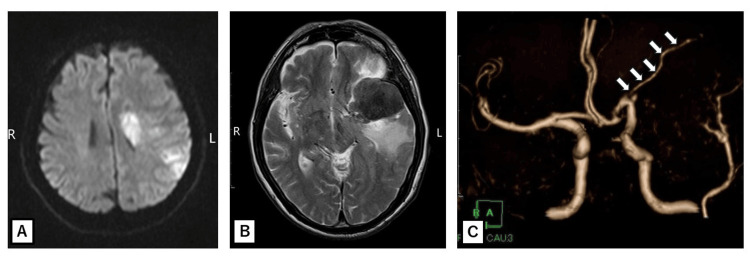
Initial brain imaging findings (A) Diffusion-weighted image demonstrating acute infarction in the left territory of the middle cerebral artery, which appeared as a hyperintense signal due to restricted diffusion. (B) A T2-weighted image revealing a well-demarcated, hypointense extra-axial mass along the left sphenoid ridge, which is consistent with meningioma, with surrounding edema involving the adjacent frontal and temporal lobes. (C) Time-of-flight magnetic resonance angiography showing complete occlusion of the proximal segment of the left middle cerebral artery. The arrows indicate the recurrent artery of Heubner.

Urgent brain imaging was performed. Diffusion-weighted MRI confirmed an acute infarction in the left MCA territory, predominantly in the parietal and temporal lobes. Additionally, a large extra-axial mass (~5 cm diameter) was observed in the left sphenoid wing region, compressing the inferolateral frontal and anterior temporal lobes. The lesion was isointense on T1 and hypointense on T2, with a dural attachment along the sphenoid ridge, which was consistent with meningioma. There was significant peritumoral edema causing a rightward midline shift. Time-of-flight MR angiography (MRA) revealed an absence of flow in the proximal M1 segment of the left MCA, indicating occlusion of the artery’s trunk, with no flow in distal MCA branches. The left anterior cerebral artery filled normally from the left internal carotid artery (which remained patent), but the MCA was displaced upward by the mass. No intratumoral hemorrhage was evident on gradient-echo sequences. These findings prompted the diagnosis of acute ischemic stroke in the left MCA territory due to extrinsic arterial compression by a sphenoid wing meningioma (Figure 1).

Given the atypical stroke etiology, conventional reperfusion therapies were reconsidered. The unknown time of onset precluded intravenous thrombolysis. Mechanical thrombectomy, while indicated for M1 occlusions in select patients up to 24 hours from the last known well, was deemed unlikely to succeed in this case because the occlusion was due to external compression rather than an intravascular embolus. Furthermore, urgent neurosurgical resection of the meningioma was shown to carry high risk in the setting of a large acute infarct with developing edema, compounded by the patient’s advanced age. After multidisciplinary consultation, the team decided to pursue intensive conservative management instead of any acute surgical or endovascular intervention. The patient was admitted to a stroke unit for close monitoring, and aggressive medical therapy was initiated to optimize cerebral perfusion and mitigate secondary brain injury. He was started on triple antiplatelet therapy (aspirin 100 mg daily, clopidogrel 75 mg daily, and intravenous ozagrel sodium 80 mg twice daily for 14 days; a thromboxane A₂ inhibitor) to inhibit thrombosis. Edaravone (30 mg twice daily for 14 days; a free radical scavenger) was administered to enhance microcirculation and reduce oxidative damage in the ischemic brain. To counter cerebral edema and elevated intracranial pressure, intravenous glycerol was given as an osmotic agent: a 10% solution, 200 mL every six hours for 10 days, followed by 200 mL every 12 hours for an additional 3 days, then discontinued. Head elevation and standard supportive measures (hydration, blood pressure control, etc.) were also employed.

**Figure 2:**
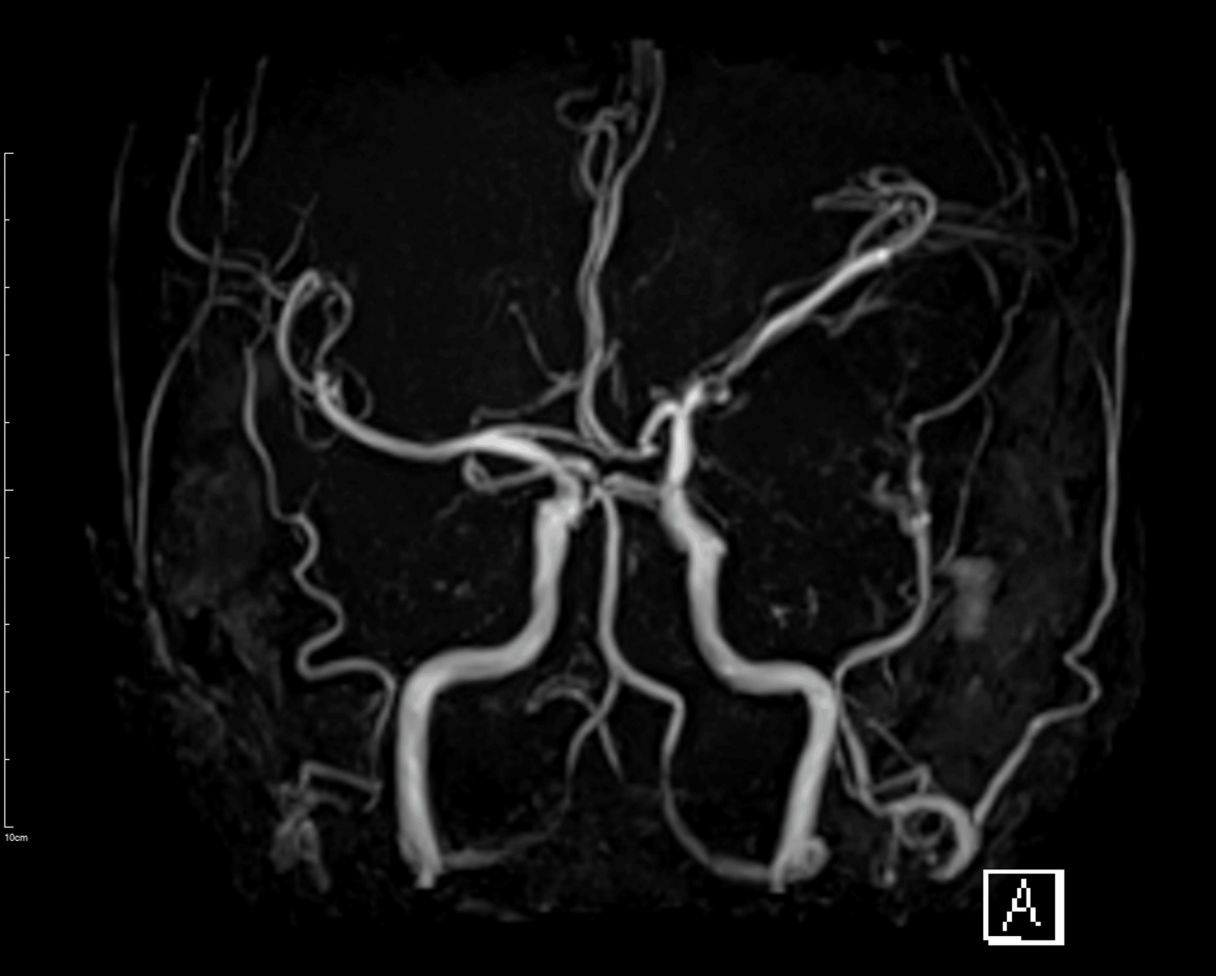
Brain angiography performed one week after onset Time-of-flight magnetic resonance angiography performed one week after stroke onset revealed recanalization of the previously occluded left middle cerebral artery with restoration of antegrade flow.

The patient’s neurological status remained stable without deterioration during the first week. A follow-up MRI on day seven revealed an established infarct in the left parietotemporal region, and T2-fluid-attenuated inversion recovery (FLAIR) imaging confirmed that the infarction remained localized (it had not progressed to involve the entire MCA territory). Importantly, MRA at one week demonstrated reestablishment of flow in the left MCA, with the previously occluded M1 segment now patent and flow visible into distal branches (Figure 2). This was interpreted as spontaneous recanalization in the subacute phase, likely due to endogenous fibrinolysis or a relief of mechanical compression as cerebral edema improved. Conservative management was continued thereafter. Clopidogrel was discontinued after 15 days, while aspirin was continued as a single antiplatelet agent for long-term secondary prevention, including throughout the post-discharge period. Following his stabilization, the patient underwent intensive rehabilitation and showed gradual neurological improvement, with partial motor and language recovery. At the time of discharge, his functional status corresponded to a modified Rankin Scale (mRS) score of four, indicating moderate-to-severe disability [5].

**Figure 3:**
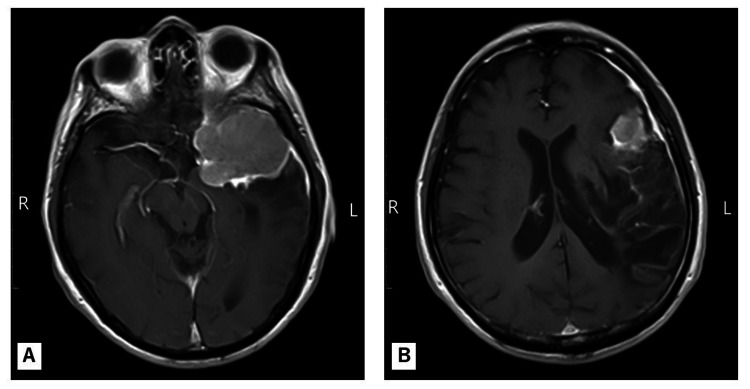
Gadolinium-enhanced axial T1-weighted brain MRI performed during the one-year follow-up (A) Homogeneous enhancement of a sphenoid ridge meningioma with a characteristic dural tail sign is observed. (B) The left frontal lobe is largely preserved, whereas the left parietal and temporal lobes show encephalomalacia and volume loss secondary to infarction.

**Figure 4:**
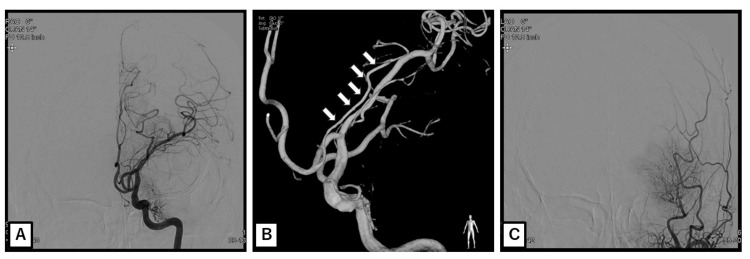
Cerebral angiography performed during the one-year follow-up (A) Digital subtraction angiography of the left internal carotid artery reveals a patent middle cerebral artery displaced superiorly by the adjacent tumor. (B) Three-dimensional digital subtraction angiography; the arrows indicate the recurrent artery of Heubner. (C) Injection of the left external carotid artery revealed a tumor blush supplied by the left middle meningeal artery.

**Figure 5:**
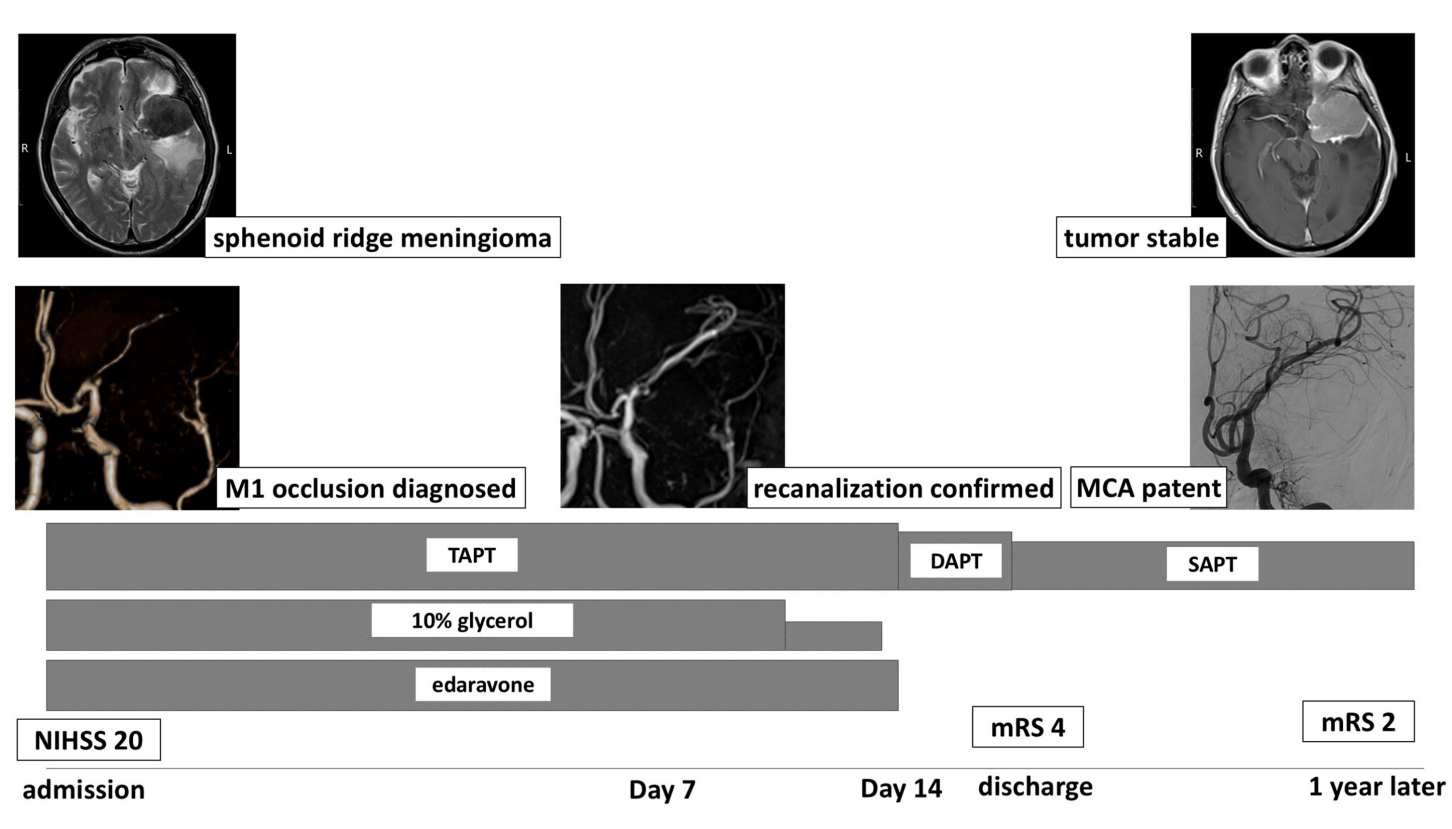
Timeline summarizing the clinical course, imaging findings, treatment milestones, and neurological outcomes Day 0 corresponds to the day of hospital admission following wake-up stroke. Follow-up MR angiography on Day 7 confirmed recanalization of the previously occluded left middle cerebral artery (MCA). Intensive medical treatment, including triple antiplatelet therapy (TAPT: aspirin, clopidogrel, and ozagrel), edaravone, and intravenous glycerol, was gradually tapered. The patient’s neurological status improved from an NIHSS score of 20 at admission to an mRS of four at discharge and an mRS of two at the one-year follow-up. Follow-up angiography and MRI at one year demonstrated sustained MCA patency and stable sphenoid wing meningioma. DAPT: dual antiplatelet therapy; SAPT: single antiplatelet therapy; NIHSS: National Institutes of Health Stroke Scale; mRS: modified Rankin Scale

After discharge, the meningioma was managed conservatively with periodic follow-up, given the patient’s age, residual deficits, and the tumor’s stable appearance. Elective tumor resection was reserved for the future if there were signs of clinical or radiological progression. At the one-year follow-up, the patient remained free of any recurrent stroke. His neurological function improved further: he was able to ambulate independently without assistance, and his aphasia improved to the point of communicating in single words (fluency was still limited). His functional status at this time corresponded to an mRS score of two [5], and aspirin therapy had been maintained throughout the follow-up period. A follow-up brain MRI with contrast at one year revealed a sphenoid ridge meningioma with homogeneous enhancement and a dural tail that was unchanged in size. Expected encephalomalacic changes (gliosis and volume loss) were present in the left parietal and temporal lobes, while the left frontal lobe was largely preserved (Figure 3). Cerebral angiography at one year confirmed sustained patency of the left MCA (now displaced upward by the adjacent tumor but fully open), as well as normal flow through the left anterior cerebral and left internal carotid arteries. An external carotid injection revealed a tumor blush from the left middle meningeal artery, which was consistent with the meningioma’s blood supply. These findings indicated that the arterial circulation had remained intact and that the tumor had not progressed (Figure 4). A timeline summarizing key clinical and imaging milestones is presented in Figure 5.

## Discussion

This case highlights an exceedingly rare phenomenon: a benign meningioma causing acute ischemic stroke by completely occluding a major cerebral artery. Only a handful of case reports (no more than 10 publications to date) have described meningioma causing major cerebral artery occlusion (see Table 1 for a summary). To our knowledge, this is the first reported instance of such a case managed successfully with medical recanalization alone.

**Table 1:** Review of meningioma-associated cerebral infarction cases in the literature ICA: internal carotid artery; MCA: middle cerebral artery; ACA: anterior cerebral artery; STA: superficial temporal artery; PCA: posterior cerebral artery; EC-IC: extracranial-intracranial; mRS: modified Rankin scale

Author (Year)	Age	Sex	Meningioma Location	Occluded Artery	Infarct Territory	Treatment	Recanalization?	Neurological Outcome
Komotar et al., 2003 (Case 1) [1]	49	M	Right cavernous sinus region	Right cavernous ICA	Right MCA territory	Focused radiotherapy on the tumor	Not stated	Initial improvement of deficits; however, a new left leg weakness occurred ~6 months later due to re-thrombosis of the ICA.
Komotar et al., 2003 (Case 2) [1]	31	M	Right olfactory groove region	Right ICA	Right MCA territory	Complete surgical resection of the tumor	Not stated	Residual deficits: permanent right eye vision loss and left arm numbness; otherwise recovered.
Heye et al., 2006 [6]	48	F	Right medial sphenoid wing/cavernous sinus	Right ICA stenosis (cavernous segment)	Right MCA territory	Endovascular stent placement in the ICA	Likely (angiographic improvement)	Partial recovery; left hemiparesis improved, with mild residual hand weakness (4th–5th fingers).
Masuoka et al., 2010 [7]	31	M	Right planum sphenoidale (frontal skull base)	Right A2 segment (ACA)	Right ACA territory	Bilateral frontal craniotomy with gross-total resection	Not stated	No new deficits postoperatively; further neurological improvement not specified in report.
Cheng et al., 2011 [8]	58	M	Right sphenoid wing (medial)	Right M1 segment (MCA)	Right MCA territory	Simpson grade III resection via pterional approach	Not stated	Persistent hemiparesis immediately post-op; after three months of rehabilitation, mild residual left arm paresis (moderate recovery).
Mathis et al., 2013 [9]	30	F	Right sphenoid wing	Right MCA (M1 segment)	Right MCA territory	Complete tumor resection	Not stated	Good recovery; regained most neurological functions; only moderate residual spasticity in right arm and leg.
Ko et al., 2014 [10]	52	M	Right sphenoid wing (medial)	Right MCA (M1) at ICA terminus	Right MCA territory	Emergency STA–MCA bypass (EC–IC double-barrel bypass)	No (revascularization by bypass)	Dramatic improvement – preoperative left-sided weakness resolved within 48 hours post-bypass.
Missori et al., 2015 [11]	48	F	Tuberculum sellae	Left A2 segment + Left recurrent artery of Heubner	Left ACA territory (cortex + caudate/internal capsule)	Tumor resection (bifrontal approach)	Not stated	Full recovery from numbness and visual symptoms; no residual tumor
Kim et al., 2016 [3]	75	F	Left petroclival region	Right PCA (P1 segment)	Right PCA territory	Fractionated radiotherapy (four courses) six years prior; no new intervention at infarct presentation	No	Outcome not detailed; presented with left homonymous hemianopia; no further clinical improvement reported.
Huang et al., 2018 [12]	63	F	Left medial sphenoid wing	Left ICA (compression of M1 segment)	Left MCA territory	Subtotal resection + EC–IC bypass (STA–MCA) + ipsilateral decompressive craniectomy	No (revascularization by bypass)	Initially had worsened right-side weakness (4/5 motor strength) post-op; full strength recovered by six months, visual acuity unchanged from pre-op.
Sharma et al., 2023 [2]	36	F	Left medial sphenoid wing (planum sphenoidale)	Left M1 segment (MCA), encasing ICA	Left MCA territory	Emergency pterional craniotomy with gross-total tumor resection (decompressive craniectomy)	No	Survived but severely disabled; persistent right hemiplegia and aphasia.
Present case, 2025	76	M	Left sphenoid wing	Left M1 segment (MCA)	Left MCA territory	Triple antiplatelet therapy + glycerol	Yes	Good recovery; Slight disability (mRS 2).

Historically, tumor-related ischemic strokes have been managed with emergency neurosurgical intervention to decompress the affected vessel [8]. Several published cases involve urgent meningioma resection (sometimes accompanied by extracranial‒intracranial bypass [10,12]) or subsequent radiotherapy [1]. However, immediate tumor removal in the context of acute infarction is highly risky and often yields suboptimal outcomes [2]. Several factors likely explain this. First, standard thrombolytic therapy or even antiplatelet agents cannot be used around the time of surgery due to bleeding risk [11], leaving any intraluminal thrombus untreated [6]. Second, the acutely infarcted brain is edematous and fragile; surgical manipulation in this state can precipitate hemorrhagic transformation or edema expansion. Revascularization surgery performed too early after stroke is associated with a high risk of hemorrhage in the infarcted territory [10]. Third, meningiomas that encase arteries often obliterate the normal arachnoid plane, making dissection extremely difficult. Attempts to peel the tumor from an adherent artery in an acutely ischemic brain risk vessel injury or rupture [8]. Finally, one must consider the impact of surgery on collateral circulation. Meningiomas grow slowly and often allow the development of collateral channels, including external carotid to dural or leptomeningeal anastomoses. Rapid surgical resection may disrupt these compensatory pathways, increasing the risk of infarction [12]. These issues likely contribute to the poor outcomes observed in some prior cases of emergent tumor resection.

Endovascular recanalization might be considered an alternative to open surgery for these unusual types of stroke, but its role remains unproven. There are no documented cases of emergency intracranial stent placement for an artery compressed by a tumor [6]. Mechanical thrombectomy alone would likely fail to reopen a vessel that is externally compressed by a mass. Heye et al. reported a case of cavernous internal carotid artery stenosis from a sphenoid wing meningioma that was treated electively with an intracranial stent; however, this was a subacute scenario after the tumor was deemed unresectable, and standard dual-antiplatelet therapy posed separate risks [6]. In acute occlusion with ongoing external compression, deploying a stent or retrieval device could risk vessel injury and would not address the underlying mechanical pressure. Thus, in our case, neither conventional surgery nor standard endovascular therapy offered an ideal solution.

The pathophysiology of ischemic stroke from a meningioma involves both hemodynamic compromise and thrombus formation. Slow-growing meningiomas often allow robust collateral circulation to develop, which is one reason many meningiomas encasing large arteries do not cause infarction. When infarction does occur, it usually results from a combination of critically reduced perfusion (for instance, during a transient hypotensive episode or increased intracranial pressure) and superimposed thrombosis at the site of compression. In our patient, we suspected that compression of the left MCA by the tumor (and swelling brain) led to intraluminal thrombosis. Similar phenomena have been reported in the literature, such as “stump” thrombus formation in a meningioma-occluded internal carotid artery [10]. Importantly, such tumor-associated thrombi may be amenable to medical dissolution or stabilization. With this in mind, we pursued an aggressive medical approach in our case to recanalize the artery. We administered triple antiplatelet therapy (aspirin, clopidogrel, and ozagrel) to maximally inhibit thrombosis and encourage arterial reopening. Notably, such potent antithrombotic therapy would have been unsafe if immediate surgery were planned, as surgical hemostasis would be compromised. By forgoing upfront surgery, we were able to safely use aggressive antithrombotics, and this was followed by a remarkable spontaneous recanalization of the MCA.

Another key element of our management was the control of intracranial pressure and edema. Poststroke cytotoxic edema, compounded by tumor-related vasogenic edema, can further exacerbate arterial compression in the fixed cranial compartment. In previous reports where meningiomas were resected shortly after a stroke, severe brain swelling was encountered intraoperatively (sometimes requiring decompressive craniectomy) [2]. We hypothesized that lowering intracranial pressure could relieve some of the external pressure on the MCA in our patient, thereby facilitating reperfusion. This concept remains hypothetical, as we lacked direct evidence that osmotherapy effectively decompressed the artery. Even so, it was a reasonable inference given the clinical circumstances. Therefore, we administered intravenous glycerol as a hyperosmolar agent to reduce cerebral edema. Osmotic therapy (e.g., glycerol or mannitol) is known to decrease intracranial pressure and may improve cerebral perfusion in acute stroke edema [13,14]. In essence, by combining thrombus dissolution and edema reduction, we aimed to restore patency in the MCA without neurosurgical intervention. The success of this strategy was supported by follow-up imaging, which confirmed reestablished flow in the MCA. Consequently, most of the initially threatened brain tissue, particularly in the anterior cerebral artery territory and frontal lobe, which are at risk, was spared from infarction, and the patient’s neurological deficits improved in parallel.

However, regardless of the triple antiplatelet therapy and glycerol we administered, one must consider that the occluded MCA might have recanalized spontaneously (or that robust collateral flow preserved perfusion) independent of our medical interventions, as spontaneous recanalization of an acute large-artery occlusion is a well-documented phenomenon in stroke patients.

That said, it is noteworthy that none of the previously reported cases of ischemic stroke caused by intracranial meningiomas documented spontaneous recanalization of the occluded vessel. In all published instances we reviewed, restoration of arterial flow required surgical or endovascular intervention (or else the occlusion persisted). For instance, Sharma et al. [2] reported a sphenoid wing meningioma that caused an M1 occlusion without spontaneous reopening. The thrombus remained lodged until it was removed by emergency craniotomy, which was complicated by severe intraoperative brain swelling that necessitated decompressive craniectomy. Similarly, Komotar et al. [1] described two meningioma stroke cases in which the arterial occlusions did not recanalize on their own; one patient’s internal carotid artery remained blocked and relied on collateral flow (with eventual stroke recurrence months later). In another report, Heye et al. [6] managed a meningioma-induced intracranial internal carotid artery stenosis by deploying an endovascular stent. This emphasized that recanalization was achieved only through active intervention rather than spontaneously. Multiple other cases in the literature echo this pattern: meningiomas (and even other tumors like high-grade gliomas) compressing or infiltrating cerebral arteries have led to infarction, but never a documented spontaneous recanalization without surgical or mechanical assistance [15,16]. In all such cases, either the vessel remained occluded until tumor resection or revascularization procedures were performed, or the patients suffered permanent arterial closure.

In contrast, Neumann-Haefelin et al. [17] reported spontaneous recanalization in six of 25 patients with MCA main stem occlusion who did not receive thrombolytic therapy. However, none of these cases involved tumor-related vascular compression. Our case, in contrast, appears unique in that the tumor-related MCA occlusion regained patency without direct mechanical intervention. To our knowledge, such a scenario has not been previously reported in the context of tumor-associated stroke.

We acknowledge that our proposed mechanism (tumor mass effect relieved by edema control) is speculative; nevertheless, the favorable outcome in this case suggests that coupling aggressive medical thrombolysis/antiplatelet therapy with intracranial pressure management can be an effective approach when conventional mechanical recanalization is risky or not feasible. The contrast with prior cases (which almost uniformly required surgical tumor removal or endovascular procedures to achieve recanalization) underscores the importance of considering a multimodal medical strategy in select patients while remaining vigilant that spontaneous recanalization or collateral perfusion might also play a role in such recovery.

Our experience differs from that of most prior cases [9] in that we did not perform early tumor removal. In the literature, definitive tumor treatment is usually undertaken, although sometimes in a delayed or staged fashion. For example, Komotar et al. [1] reported one case in which radiosurgery (followed by anticoagulation) was performed for a meningioma encasing the carotid artery and another case in which an olfactory groove meningioma causing stroke was treated with craniotomy. Masuoka et al. [7] described a patient who underwent delayed craniotomy three months after a stroke caused by a small sphenoid meningioma once the patient’s condition stabilized. Taken together, these reports imply that early elective tumor resection following stabilization may be preferable in younger, medically fit patients, as it could mitigate the risk of recurrent infarction or progressive tumor-related symptoms. In our patient, by achieving medical recanalization, we were able to defer any immediate intervention on the tumor. Additionally, the patient’s advanced age and personal preference to focus on neurological rehabilitation (rather than undergo immediate surgery) influenced our decision to postpone tumor resection. We anticipated that the infarcted brain tissue would gradually atrophy, reducing intracranial pressure, and that delaying surgery would allow time to safely discontinue antiplatelet therapy before craniotomy. For these reasons, we managed the tumor conservatively for over a year without resection, and we plan to cease antiplatelet medication and proceed with an elective craniotomy for tumor removal once conditions are optimized. Not all instances of tumor-related arterial occlusion necessarily mandate emergent surgery, especially if the surgical risk is exceedingly high (for example, in an elderly patient with significant comorbidities). This case demonstrates that, in select scenarios, an initial trial of medical management can stabilize the patient and even reverse vascular occlusion, allowing for tumor treatment later in an elective, controlled setting. Elective resection or irradiation of the tumor can be pursued once the stroke has stabilized.

From this, a broader management algorithm can be inferred for stroke caused by meningioma. In patients who are not in immediate life-threatening danger from mass effects, a trial of intensive medical therapy could be considered to reopen the artery. This includes optimizing cerebral perfusion (e.g., head elevation, hyperosmolar therapy, blood pressure support) and targeting the thrombus component (with anticoagulation or multiagent antiplatelet therapy) while closely monitoring for any deterioration. If the patient’s neurological status or subjective symptoms (such as headache or nausea) worsen or fail to improve despite intensive medical therapy, urgent surgical intervention (such as craniotomy for tumor resection and decompression) would be warranted as the next step [18]. If signs of improvement and vessel recanalization are observed, one can then proceed with definitive tumor-directed treatment electively when perioperative antithrombotic management can be adjusted appropriately (e.g., holding antiplatelets prior to surgery). Such a staged strategy can be especially prudent in an older patient or one with significant comorbidities, as it allows time for neurological recovery and ensures safer elective tumor resection once antithrombotic therapy is discontinued. Unfortunately, neither CT nor MR perfusion imaging was performed at the time of the patient’s stroke, which represents a limitation of this report. Consequently, the decision to pursue medical management was based on clinical judgment and conventional MRI/MRA findings. Perfusion imaging could have been valuable in demonstrating salvageable penumbra and supporting the rationale for conservative treatment; its availability might have further informed therapeutic decision-making.

Finally, one must remain cognizant that leaving a meningioma untreated carries long-term risks [19]. Even histologically benign meningiomas may gradually enlarge over time, underscoring the importance of serial imaging follow-up [20]. Furthermore, persistent tumor presence can lead to recurrent ischemic events: Komotar et al. [1] reported a case of recurrent stroke six months after initial presentation despite radiotherapy, and Masuoka et al. [7] described a patient whose initial transient ischemic attacks progressed to a cerebral infarction prior to tumor resection. Therefore, vigilant long-term surveillance and timely elective intervention are critical to mitigate these risks.

## Conclusions

This case illustrates that when a meningioma causes acute large-artery occlusion, intensive medical therapy may not only stabilize the patient but also achieve recanalization of the occluded vessel. This, in turn, may eliminate the need for immediate surgical intervention. In appropriately selected patients, a nonsurgical therapeutic trial can be a viable alternative to emergent craniotomy, allowing definitive tumor treatment to be safely deferred to a later, elective setting. This approach may help avoid the heightened risks associated with urgent tumor resection in the context of an acute infarction. Prospective studies are needed to determine which patients are most likely to benefit from this strategy.

## References

[1] Komotar RJ, Keswani SC, Wityk RJ (2003). Meningioma presenting as stroke: report of two cases and estimation of incidence. J Neurol Neurosurg Psychiatry.

[2] Sharma GR, Paudel P, Karki P (2023). Meningioma presenting as ischemic stroke: case report and review of literature. Neurosurg Cases Rev.

[3] Kim BJ, Lee DH, Kang DW (2016). Petroclival meningioma accompanying posterior cerebral artery infarction. J Stroke.

[4] van Swieten JC, Koudstaal PJ, Visser MC, Schouten HJ, van Gijn J (1988). Interobserver agreement for the assessment of handicap in stroke patients. Stroke.

[5] Brott T, Adams HP Jr, Olinger CP (1989). Measurements of acute cerebral infarction: a clinical examination scale. Stroke.

[6] Heye S, Maleux G, Van Loon J, Wilms G (2006). Symptomatic stenosis of the cavernous ICA due to an irresectable sphenoid wing meningioma: treatment by endovascular stent placement. AJNR Am J Neuroradiol.

[7] Masuoka J, Yoshioka F, Ohgushi H, Kawashima M, Matsushima T (2010). Meningioma manifesting as cerebral infarction. Neurol Med Chir (Tokyo).

[8] Cheng HT, Wang CC, Chio CC, Kuo JR (2011). Sphenoid ridge meningioma presenting as ischemia stroke. ANZ J Surg.

[9] Mathis S, Bataille B, Boucebci S, Jeantet M, Ciron J, Vandamme L, Neau JP (2013). A rare cause of stroke in young adults: occlusion of the middle cerebral artery by a meningioma postpartum. Case Rep Neurol Med.

[10] Ko JK, Cha SH, Choi CH (2014). Sphenoid ridge meningioma presenting as acute cerebral infarction. J Korean Neurosurg Soc.

[11] Missori P, Morselli C, Fattapposta F, Peschillo S, Currà A (2015). Ischaemic stroke with partial haemorrhagic transformation related to a small-sized tuberculum sellae meningioma. Neurol Sci.

[12] Huang Y, Wang Z, Han Q (2018). Extracranial-intracranial bypass in medial sphenoid ridge meningioma associated with severe stenosis of the intracranial segments of the internal carotid artery: a case report. Medicine (Baltimore).

[13] Chen JL, Wang YC, Wang JY (1989). Haemodynamic and cerebrovascular responses to glycerol infusion in dogs. Clin Sci (Lond).

[14] Wang JY, Chen JL (1994). Glycerol dehydrates oedematous as well as normal brain in dogs. Clin Exp Pharmacol Physiol.

[15] Kasapas K, Malli A, Kassioti E, Valkimadi PE (2020). Posterior circulation ischemic stroke secondary to high-grade glioma: a rare case report and review of the literature. Cureus.

[16] Züchner S, Kawohl W, Sellhaus B, Mull M, Mayfrank L, Kosinski CM (2003). A case of gliosarcoma appearing as ischaemic stroke. J Neurol Neurosurg Psychiatry.

[17] Neumann-Haefelin T, du Mesnil de Rochemont R, Fiebach JB (2004). Effect of incomplete (spontaneous and postthrombolytic) recanalization after middle cerebral artery occlusion: a magnetic resonance imaging study. Stroke.

[18] (2018). Headache Classification Committee of the International Headache Society (IHS) the International Classification of Headache Disorders, 3rd edition. Cephalalgia.

[19] Nakasu S, Nakasu Y (2020). Natural history of meningiomas: review with meta-analyses. Neurol Med Chir (Tokyo).

[20] Maggio I, Franceschi E, Tosoni A, Nunno VD, Gatto L, Lodi R, Brandes AA (2021). Meningioma: not always a benign tumor. A review of advances in the treatment of meningiomas. CNS Oncol.

